# A Potential Application of *Pseudomonas psychrotolerans* IALR632 for Lettuce Growth Promotion in Hydroponics

**DOI:** 10.3390/microorganisms11020376

**Published:** 2023-02-02

**Authors:** Chuansheng Mei, Dongfang Zhou, Robert L. Chretien, Amy Turner, Guichuan Hou, Michael R. Evans, Scott Lowman

**Affiliations:** 1 Plant Endophyte Research Center, Institute for Advanced Learning and Research, Danville, VA 24540, USA; 2 School of Plant and Environmental Sciences, Virginia Tech, Blacksburg, VA 24061, USA; 3 Dewel Microscopy Facility, College of Arts and Sciences, Appalachian State University, Boone, NC 28608, USA

**Keywords:** plant growth-promoting bacteria (PGPB), bacterial endophytes, *Lactuca sativa*, nutrient film technique, Green Oakleaf

## Abstract

Controlled environment agriculture hydroponic systems grow plants year-round without restriction from outside environmental conditions. In order to further improve crop yield, plant growth-promoting bacteria were tested on hydroponically grown lettuce (*Lactuca sativa*) plants. From our bacterial endophyte library, we found one bacterium, *Pseudomonas psychrotolerans* IALR632, that is promising in promoting lettuce growth in multiple hydroponic systems. When Green Oakleaf lettuce seeds were inoculated with IALR632 during germination, IALR632 significantly increased lateral root development by 164%. When germinated seedlings were inoculated with IALR632 and then transplanted to different hydroponic systems, shoot and root fresh weights of Green Oakleaf increased by 55.3% and 17.2% in a nutrient film technique (NFT) system in the greenhouse, 13.5% and 13.8% in an indoor vertical NFT system, and 15.3% and 13.6% in a deep water cultivation (DWC) system, respectively. IALR632 also significantly increased shoot fresh weights of Rex by 33.9%, Red Oakleaf by 21.0%, Red Sweet Crisp by 15.2%, and Nancy by 29.9%, as well as Red Rosie by 8.6% (no significant difference). Inoculation of IALR632-GFP and subsequent analysis by confocal microscopy demonstrated the endophytic nature and translocation from roots to shoots. The results indicate that *P. psychrotolerans* IALR632 has a potential application in hydroponically grown lettuce plants.

## 1. Introduction

The world population will increase to as much as 9.5–10 billion by 2050. In order to sustainably feed the increasing number of people, food yield needs to increase by 70% [1]. Controlled environment hydroponic systems can supplement typically field-grown crops year-round without limitation or restriction by biotic and abiotic stresses. The use of pesticides is also eliminated in many cases, responding to consumer demand for herbicide and pesticide-free food. Various systems have been developed to increase the quality and growth rate of plants [2]. These systems can provide both high-yield and high-quality agricultural crops on demand. Hydroponic systems have many advantages, such as water and nutrient reuse, easy control of the environmental conditions, and the prevention of soil-borne diseases and pests [3]. In addition, the beneficial effects on the environment are substantial, such as up to 95% water savings [4], less pesticide and fertilizer use, 90% less land use, and a potential reduction in carbon emissions by 70% in green vertical farming [5]. In the past 5 years, the number of commercial hydroponic industries has dramatically increased. Research and markets report that the global indoor farming market was valued at USD 14.3 billion in 2017 and is expected to reach USD 24.9 billion by 2027 at a compound annual growth rate of 5.7% [6]. 

The hydroponically grown produce market has grown dramatically in recent years, and associated technologies have also been greatly improved, including fine-tuned plant growth nutrient mixtures, light intensity and quality, and temperature [7]. However, as plants grow, they change the chemical composition of the nutrient solution by depleting specific nutrients more rapidly than others, removing water from the solution and altering the pH. Therefore, optimizing conditions for maximal plant growth is a focus in commercial hydroponic production systems. It is worthwhile to study how to maintain plant growth sustainably, even in suboptimal conditions. One alternative solution for increasing growth is to use beneficial plant growth-promoting bacteria (PGPB). PGPB, including soil, rhizosphere, epiphytic, and endophytic bacteria, can help plants achieve optimal growth, even in suboptimal conditions [8]. Overall, PGPB can promote plant growth and yield, enhance nutrient uptake, and increase stress tolerance, as well as fight against diseases [9,10,11,12]. Most mechanisms for plant growth promotion by PGPB have been elucidated. Some can produce the plant growth-promoting hormone auxin, which increases plant root size and enhances uptake of nutrients. Some have high levels of 1-aminocyclopropane-1-carboxylate (ACC) deaminase activity, which breaks down ACC, a precursor in the biosynthesis pathway of ethylene, a plant growth-inhibiting hormone [10]. Lowering ethylene levels can enhance plant tolerance to abiotic stresses [10]. Others may solubilize phosphate in soil or fix nitrogen from the atmosphere. There have been a number of PGPB used in hydroponic systems [2,13,14]. For example, Paradiso et al. [15] reported that plant growth-promoting microorganisms enhanced the photosynthesis activity and seed yield of soybean plants grown in hydroponic systems. Kanjanamaneesathian et al. [16] sprayed the roots of lettuce plants with *Bacillus velezensis* solutions and increased root and shoot growth. Moncada et al. [17] reported that a commercial biostimulant product (TNC Bactorr^S13^ containing *Bacillus* spp.) increased lettuce growth and minimized salt stress in a floating system. In particular, *Pseudomonas* is a varied genus of Gram-negative, rod-shaped, motile, aerobic ɤ-proteobacteria, and is widely distributed in complicated lineages and phylogenetic groups. Beneficial *Pseudomonas* spp. have various functions, including plant growth promotion and abiotic stress tolerance, as well as pathogen growth inhibition [18]. For example, *Pseudomonas* sp. LSW25R promoted tomato plant growth, increased Ca uptake, and inhibited diseases in a hydroponic system [19]. *Pseudomonas chlororaphis*-treated romaine lettuce were taller and produced more biomass than those of control plants in a window hydroponic system [20]. 

Recently, we reported plant growth promotion in vitro and in the greenhouse by five phosphate-solubilizing bacterial endophytes [21]. One of the bacteria, *Pseudomonas psychrotolerans* IALR632, was isolated from leaves of *Sorghum halepense*, and its 16S rRNA sequence was deposited in NCBI with accession number MZ519967 [21]. IALR632 has several plant growth-promoting traits, including plant growth hormone and siderophore production, N fixation, and ACC deaminase activity. It demonstrated growth promotion of pepper and tomato in greenhouse experiments with insoluble phosphate compounds [21]. Since Green Oakleaf is a cultivar in the Salanova group of lettuce cultivars specifically bred for use in indoor agriculture [22], the objectives of this research were to study effects of bacterial endophyte *P. psychrotolerans* IALR632 on the growth of lettuce cv. Green Oakleaf in three different hydroponic systems (greenhouse NFT, indoor vertical NFT, and greenhouse DWC units), as well as on the growth promotion of other lettuce cultivars. We discuss possible applications of IALR632 in hydroponically grown lettuce plants. 

## 2. Materials and Methods

### 2.1. Plant Materials and Bacterial Cultures

#### 2.1.1. Lettuce Seeds and Germination

Six seed-coated lettuce (*Lactuca sativa*) cultivars were used in this study, i.e., butterhead type: Nancy and Rex; leafy type: Salanova Red Oakleaf, Salanova Green Oakleaf, Red Sweet Crisp (RSC); and romaine type: Red Rosie. These seeds were purchased from Johnny’s Selected Seeds (Fairfield, ME, USA). Among them, Salanova Green Oakleaf was used in all experiments except an experiment for other cultivars. Lettuce seeds were sowed in Oasis Grower Solutions Horticubes XL Foam Medium (276 cells) and placed in propagation systems from AmHydro (Arcata, CA, USA). The seeds were irrigated with water until germination under natural light in greenhouse. After germination, the seedlings were irrigated for 1 min 4 times daily with electrical conductivity (EC) 1.0 ± 0.1 mS/cm of the Virginia Tech fertilizer solution (see Section 2.3.1 for each ingredient) at pH 5.9 ± 0.1. The EC and pH were monitored with Economy pH/EC Meter (Spectrum Technologies, Inc., Aurora, IL, USA) and adjusted as needed. The seedlings were ready for bacterial inoculation one week after the seeds were sowed. 

#### 2.1.2. Bacterial Origin and Culture and Inoculation

*Pseudomonas psychrotolerans* IALR632 was isolated from leaves of *Sorghum halepense* grown in the foothills of the Appalachian Mountains in Central Virginia, USA (geographic location: 37.125372, −79.298415), and its 16S rRNA sequence was deposited in NCBI with accession number MZ519967 [21]. The glycerol stock was made after isolation and kept at −80 °C freezer for long-term storage. A loop of bacterial glycerol stock was taken and cultured in 4 mL of LB broth at 200 rpm at 30 °C overnight. One mL of the overnight culture was transferred to a flask with 20 mL of fresh medium and grown at 30 °C at 200 rpm for about 5 h until the OD_600_ was approximately 1.0 [1.85 × 10^9^ colony-forming units (CFUs) per mL].

#### 2.1.3. Bacterial Inoculation of Seeds

For lateral root development experiment, the bacterial culture was centrifuged at 5000 rpm for 5 min and the bacterial pellet was resuspended in PBS (phosphate-buffered saline (PBS: 137 mM NaCl, 2.7 mM KCl, 10 mM Na_2_HPO_4_, and 1.8 mM KH_2_PO_4_) and diluted to an OD_600_ of 1.0 for inoculation. Three Petri dishes (20 seeds/dish) were incubated with 5 mL of bacterial solution in PBS and grown at 20 °C for 12 h light/12 h dark in an incubator for 10 days. Seeds were incubated with 5 mL of PBS only as the control. Pelleted Green Oakleaf seeds had 100% germination rate.

#### 2.1.4. Bacterial Inoculation of Seedlings

After about one week, the seedlings were ready for bacterial inoculation. One mL of bacterial culture (OD_600_ of about 1.0) was applied to the root area of each seedling in propagation systems. The control seedlings were applied with 1 mL of noninoculated LB broth. One week post bacterial inoculation, lettuce seedlings at 3–4 true leaf stage were transplanted to hydroponic systems.

### 2.2. Hydroponic Systems

#### 2.2.1. Greenhouse NFT System

The NFT production system from CropKing (Lodi, OH, USA) was used for lettuce growth in the greenhouse. Each NFT unit (Model # NFT0406) had 6 growing channels for 6 plugs (36 plant capacity). The dimensions (in cm) of the NFT systems were 139.7 length × 139.7 width × 78.74 height, and they were raised 1 cm for every 40 cm in length (2.5% slope). 

#### 2.2.2. Vertical Indoor NFT System

The indoor HydroCycle vertical NFT lettuce and herb systems (3 levels with 48 plants/level) from FarmTek (Dyersville, IA, USA) was used. The slope of all NFT systems was set to 2.5–3%. Light was supplied with GE Arize^TM^ Lynk and Life 1.5 HO Horticulture Batten LED Luminaire (Hort Americas, Bedford, TX, USA). The lights were fixed 30 cm above the NFT surface. The CO_2_ level was maintained at 350–500 ppm.

#### 2.2.3. Deep Water Cultivation (DWC)

VEVOR Hydroponic Grow Kits (36 plants/unit, 4 channels, 1 level) (www.vevor.com, accessed on 1 February 2023) were modified to halve the capacity (18 plants/unit) and double the space between channels for lettuce growth.

### 2.3. Plant Growth Nutrient Solutions and Environments

#### 2.3.1. Lettuce Growth Nutrient Solution

The vegetative hydroponic fertilizer solution was developed at Virginia Tech (Blacksburg, VA, USA) and consists of two 100×stock solutions: Stock A (L)—90 g calcium nitrate [Ca(NO_3_)_2_], 40 g potassium nitrate (KNO_3_), and 7.54 g iron chelate (6%)–Sprint 138 (6% Chelated EDDHA Iron); and Stock B (L)—17 g potassium sulfate (K_2_SO_4_), 15 g monopotassium phosphate (KH_2_PO_4_), 60 g magnesium sulfate heptahydrate (MgSO_4_ ·7H_2_O), 310 mg manganese sulfate monohydrate (MnSO_4_ · H_2_O), 30 mg zinc sulfate heptahydrate (ZnSO_4_ · 7H_2_O), 275 mg boric acid (H_3_BO_3_), 39 mg copper sulfate pentahydrate (CuSO_4_ · 5H_2_O), and 11.1 mg ammonium molybdate tetrahydrate [(NH_4_)_6_Mo_7_O_24_ · 4H_2_O]. The fertilizer solution does not account for mineral elements present in water source. Equal amounts of Stocks A and B were used to create a final dilute solution (1:90) of EC 1.5 mS/cm and adjusted to pH 5.9. The EC was adjusted by adding nutrient solution or water. The pH was adjusted by adding 1 N H_2_SO_4_ or 1 N KOH as needed. 

#### 2.3.2. Growth Conditions with WatchDog Records

The growth conditions were recorded with WatchDog 2475 Plant Growth Stations (Spectrum Technologies, Inc, Aurora, IL, USA), and data analysis was performed with SpecWare software version 9 (Table 1).

### 2.4. Transplanting and Plant Growth Measurements

#### 2.4.1. Growth Promotion

Approximately 4–7 d after bacterial inoculation, the seedlings at the 3–4 true leaf stage were transplanted to hydroponic systems. After around 3 weeks, plants were harvested. Shoot and root fresh weights (FW) were measured. Dry weights (DW) were determined after shoots and roots were dried at 60 °C for 2 d.

#### 2.4.2. Growth Rates and SPAD

After transplanting, 8 plants were taken every 4 d for 28 d to measure shoot FW and chlorophyll contents with Single-Photon Avalanche Diode (SPAD 502 Plus Chlorophyll Meter, Spectrum Technologies, Inc., Aurora, IL, USA). SPAD was measured by averaging 3 representative leaves per plant from a total of 8 plants per time point. The data were plotted with shoot FW or SPAD during growth period to compare growth rates and SPAD between the control and bacterial treatment.

### 2.5. Bacterial Colonization and Translocation

#### 2.5.1. Transformation of IALR632 with GFP Plasmid

To visualize colonization and translocation of IALR632 in lettuce plants, IALR632 wild type was transformed with plasmid p519ngfp (ATCC^®^ Cat. No. 87453, Manassas, VA, USA) using electroporation. An overnight culture of IALR632 was inoculated to half-strength LB and grown to an OD_600_ of 0.9 at 30 °C for about 4 h. The cells were cooled on ice for 10 min and harvested by centrifugation at 5000 rpm at 4 °C for 10 min. The cells were washed 4 times with ice-cold electroporation medium (10% glycerol, 0.25 M sorbitol and 0.25 M mannitol), centrifuged, and resuspended in the same medium. For electroporation, 50 ng DNA of plasmid p519ngfp was added to 1 mm gap cuvette containing 60 µL electrocompetent cells on ice, mixed by pipetting, and incubated for 1 min. The exponential protocol of the electroporator was 2500 V, 25 µF and 200 Ω with Gene Pulser Xcell^TM^ Electroporation System (Bio-Rad, Hercules, CA, USA). After electroporation, 900 µL of half-strength LB was added to the cuvette, transferred to a microcentrifuge tube, and incubated at 30 °C at 150 rpm for 2 h. The bacteria were then collected by centrifugation at 10,000 rpm for 2 min, resuspended in 200 µL, and spread on half-strength LB agar plates with 30 µg/mL kanamycin (Sigma-Aldrich, St. Louis, MO, USA). The colonies with GFP were chosen under a fluorescent microscope Olympus SZX12 (Tokyo, Japan) and named IALR632-GFP.

#### 2.5.2. Bacterial Colonization of Lettuce Plants

To remove the pellet, seeds of Green Oakleaf were vortexed in water with a drop of Tween 20 for 30 min. The residue from the pellet was then flushed through a sieve. The seeds were placed in 1% NaOCl (Clorox, Oakland, CA, USA), inverted for 15 m, and then rinsed with sterile water 4 times. The seeds were transferred to Petri dishes with sterile cellulose filter paper (Whatman^TM^, Grade 1, GE Healthcare Life Sciences, made in China) moistened with 5 mL of sterile water, and germinated for 7 d. Prior to inoculation, the seedling taproot was cut in half from root tips with a sterile scalpel. IALR632-GFP was grown to an OD_600_ of 0.5–1.0, pelleted, and then resuspended in half-strength (1/2) M519 medium (PhytoTechnology Laboratories, Lenexa, KS, USA) and incubated for 5 min (the control was incubated with 1/2 M519 medium without bacteria). The seedlings were then transferred to M519 medium with 3 g/L phytagel (no sucrose), pH 5.8. At day 9, the bacterial colonization of the roots was observed under Zeiss LSM 880 confocal workstation with Airyscan (Carl Zeiss Microscopy, LLC, Oberkochen, Germany). To check for colonization, the roots and shoots were separated at day 10, and 3 roots and 3 shoots were pooled together and weighed on analytical balance, then washed in water containing a drop of Tween 20 and inverted for 1 h, then rinsed briefly with 70% ethanol and washed 4 times with sterile water. Both roots and shoots were ground individually in 1.5 mL of sterile water. The ground materials were centrifuged at 3000 rpm for 2 min and the supernatant diluted to 1:10, 1:100, and 1:1000 with sterile water. A total of 100 µL of the diluted supernatant was plated on 1/2 LB medium containing 30 mg/L kanamycin and grown in an incubator at 30 °C overnight. The CFUs from the 1:1000 dilution were counted under fluorescence microscope Olympus SZX12. The average CFUs were from triplicate biological samples.

### 2.6. Experiment Design and Statistical Analysis

Experiments of lettuce plants in hydroponic systems were block designs, with 3 blocks and 12 plants/treatment/block for greenhouse NFTs and indoor vertical NFTs, and 9 plants/treatment/block for DWC units. Data for growth promotion by bacterial treatments were compared with the control treatment using ANOVA (JMP 16, Cary, NC, USA) for statistical analysis. Values were assigned to each group and reported at 95%, 99%, or 99.9% confidence with asterisks *, **, and ***, respectively. 

## 3. Results

### 3.1. Effects of IALR632 on Lateral Root Development

**Figure 1:**
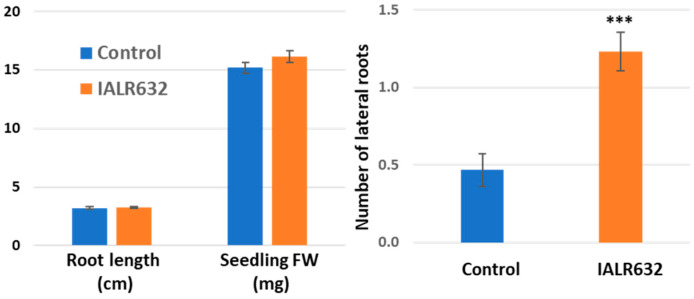
Effect of IALR632 inoculation on seedling growth and lateral root development. *** on the column means statistically significant difference at *p* < 0.001.

Ten days after seeds were incubated with IALR632 culture, the bacterial treatment only slightly increased the root length (1.9%) and fresh weight (6.4%) of seedlings. However, it greatly promoted the Green Oakleaf cultivar’s lateral root development by 164% over the controls (Figure 1).

### 3.2. Growth Promotion by IALR632 in Greenhouse NFT System

**Figure 2:**
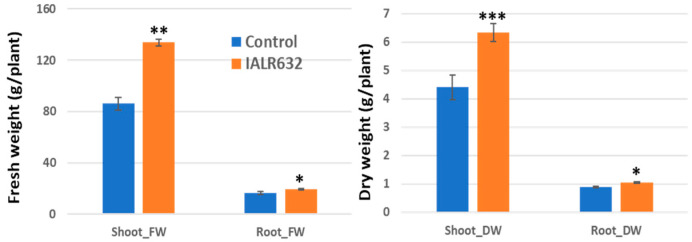
Effects of IALR632 inoculation on Green Oakleaf plant growth in greenhouse NFTs (12 April 2021 to 4 May 2021). *, **, *** on the columns mean statistically significant difference at *p* < 0.05, 0.01 and 0.001, respectively.

The inoculation of IALR632 significantly promoted Green Oakleaf plant growth, with a 55% and 44% increase in shoot fresh and dry weights and a 17% increase in both root fresh and dry weights over the control, respectively (Figure 2).

### 3.3. Effects of IALR632 on Growth Rates and SPAD in Greenhouse NFT System

**Figure 3:**
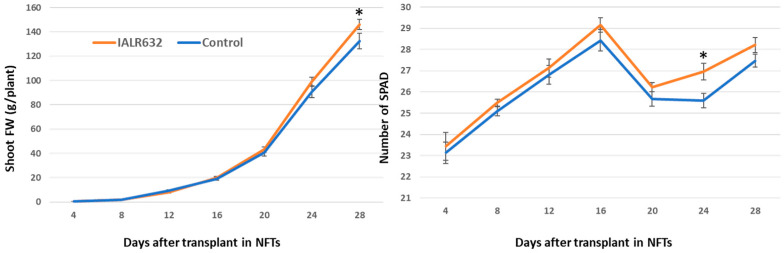
Effects of IALR632 inoculation on Green Oakleaf lettuce growth rates and SPAD for 4 weeks (23 September 2021 to 21 October 2021). * on the column means statistically significant difference at *p* < 0.05.

In the bacterial treatment, the shoot FW became greater after 16 days and was significantly higher at day 28 over the control. Plants with bacterial inoculation showed higher SPAD values than the controls from the beginning and became statistically significant at day 24 after transplant (Figure 3).

### 3.4. Growth Promotion of Other Cultivars by IALR632 in Greenhouse NFTs

**Figure 4:**
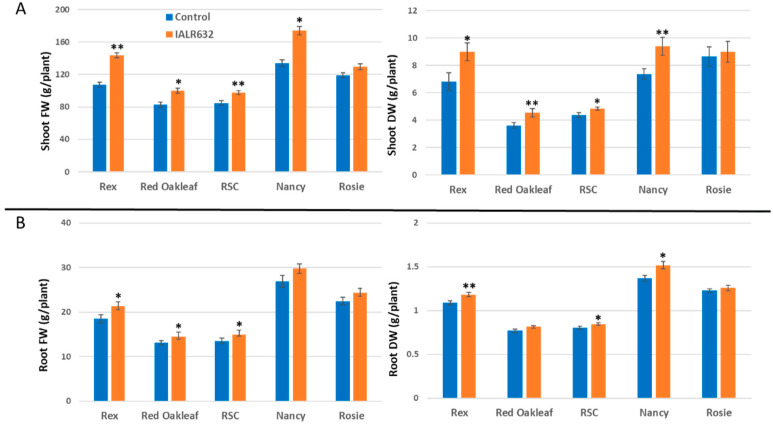
Effects of IALR632 on shoot (**A**) and root (**B**) growth of 5 cultivars in greenhouse NFT system (12 April 2021 to 4 May 2021). *, ** on the column mean statistically significant difference at *p* < 0.05 and 0.01, respectively.

IALR632 inoculation significantly enhanced the shoot fresh and dry weights of all the cultivars except Red Rosie. In Figure 4A, the shoot FW in bacterial treatment was 33.9%, 21.0%, 15.2%, and 29.9% higher than that of the control in Rex, Red Oakleaf, Red Sweet Crisp (RSC), and Nancy, respectively. The shoot DW in bacterial treatment had similar results to the shoot FW. In Figure 4B, IALR632 treatment also increased root fresh and dry weight, i.e., the root FW was 15.0%, 10.0%, 10.6%, and 10.9% higher than that of the control in Rex, Red Oakleaf, RSC, and Nancy, respectively.

### 3.5. Growth Promotion by IALR632 in Other Hydroponic Systems

#### 3.5.1. Indoor Vertical NFT Hydroponic System

**Figure 5:**
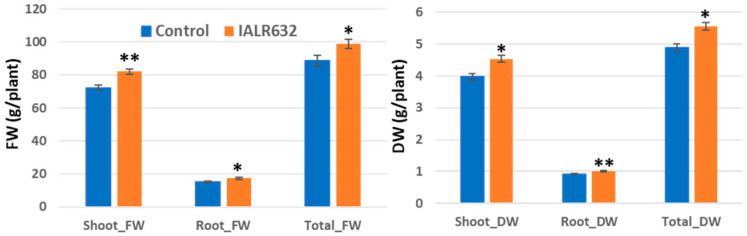
Effects of IALR632 inoculation on plant growth in indoor vertical NFTs (31 March 2002 to 22 April 2022). *, ** on the column mean statistically significant difference at *p* < 0.05 and 0.01, respectively.

Both shoot and root fresh weights and dry weights were significantly higher in bacterially inoculated plants than those of control treatments (Figure 5). Shoot and root fresh weights were increased by 13.5% and 13.8%, and shoot and root dry weight by 14.4% and 9.6%, in bacterial treatment over the control, respectively.

#### 3.5.2. Deep Water Cultivation (DWC) in Greenhouse

**Figure 6:**
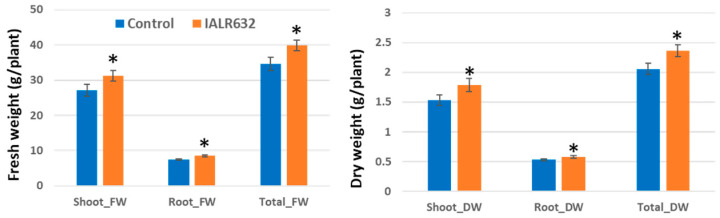
Effects of IALR632 inoculation on growth of Green Oakleaf lettuce in DWC hydroponic system in greenhouse (26 January 2022 to 16 February 2022). * on the column means statistically significant difference at *p* < 0.05.

Similar results were obtained for growth promotion of Green Oakleaf by bacterial inoculation in DWC units (Figure 6). IALR632 significantly increased the shoot and root fresh weight by 15.3% and 13.6% and the shoot and root dry weights by 16.9% and 9.7% over the controls, respectively.

### 3.6. Colonization and Localization of IALR632-GFP in Lettuce Plants

**Figure 7:**
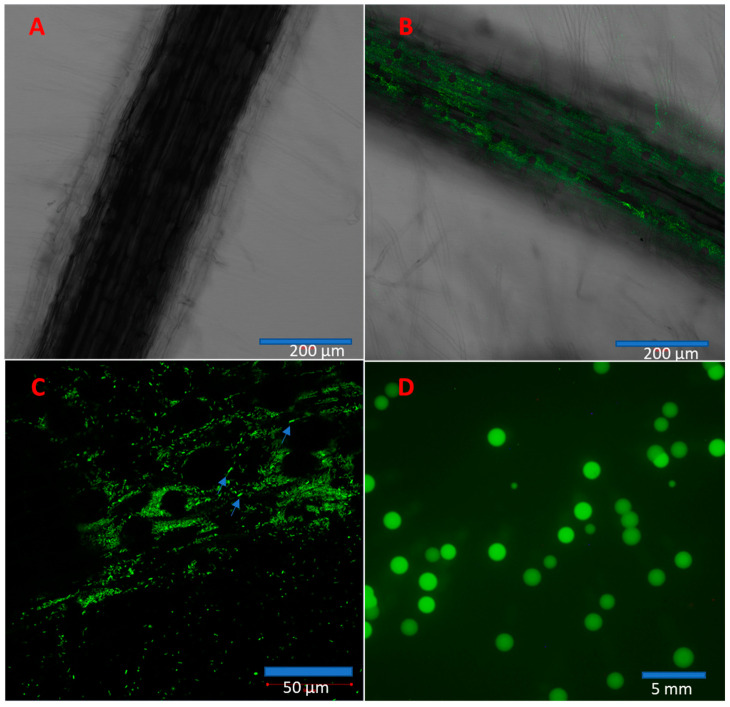
Colonization and translocation of IALR632-GFP in lettuce plants. (**A**) Confocal image from the control root with 10× magnification. (**B**) A confocal image of the root inoculated with IALR632-GFP showing green fluorescence with 10× magnification. (**C**) The same root shown in B with 40× magnification showing fluorescent bacteria (arrows). (**D**) Colonies of IALR632-GFP reisolated from leaves of seedlings inoculated with IALR632-GFP bacteria on plates under fluorescence microscope Olympus SZX12.

From confocal images, we found that the control roots did not have fluorescent bacteria (Figure 7A) while IALR632-GFP-inoculated roots did at day 9 (Figure 7B). At 40× magnification, many short rods of IALR632-GFP bacteria could be seen (blue arrows in Figure 7C). Bacteria were reisolated from root and shoot samples 10 d after bacterial inoculation. The fluorescent bacterial colonies from leaf samples were grown in plates from IALR632-GFP inoculated seedlings, which indicated bacterial translocation from roots to leaves in plants (Figure 7D). The average CFUs per mg fresh weight of roots and leaves were 1.01 ×10^5^ ± 6.75 ×10^3^ and 3.87 ×10^4^ ± 9.04 ×10^3^, respectively. 

## 4. Discussion

Studies on PGPB in hydroponically grown crops are limited. The efficacy from previous reports of PGPB in controlled environment agriculture is much higher than that in open environmental conditions where many factors are uncertain or interfere. Our studies showed that IALR632 not only significantly increased the growth of lettuce cv. Green Oakleaf in NFT (Figure 2), but also four other cultivars (Figure 4). In addition, IALR632 significantly promoted lettuce cv. Green Oakleaf growth in other hydroponic systems, including indoor vertical NFT (Figure 5) and DWC units (Figure 6). Beneficial microorganisms, such as *Bacillus*, *Pseudomonas*, and *Enterobacter,* have been studied in hydroponic systems, and some have been shown to effectively promote plant growth [2,20]. In particular, 10 different species of *Pseudomonas* from various host plants were conducted in hydroponic systems [2]. In another report, *Pseudomonas chlororaphis*-treated romaine lettuces were longer and heavier over the control plants [20]. 

The cultivar Green Oakleaf is specifically bred for controlled environment agriculture [22]. The mechanisms for this cultivar and other lettuce growth promotion by IALR632 may be complex and could be attributed to multiple factors, as the bacterium can produce plant growth-promoting hormones and siderophores, solubilize phosphate, and has ACC deaminase activity and the ability to fix nitrogen from the atmosphere [21]. The inoculation of IALR632 significantly increased lateral root development (Figure 1), which may be related to auxin production of this bacterium, as well as ACC deaminase activity of the bacterium [21], since the lateral root development is regulated by cytokinin, auxin, and ethylene [23]. Therefore, ACC deaminase production by IALR632 may also be one of the mechanisms for growth promotion. A recent review described how PGPB help increase stress tolerance by releasing metabolites or compounds such as plant hormones (auxins, gibberellins, and cytokinins) and organic acids such as succinic acid and salicylic acid [24]. In addition, exopolysaccharide accumulation helps bacterial survival and the communication between microbe–microbe and microbe–host [24]. IALR632 has a high phosphate-solubilizing ability and produces a high level of siderophore compounds [21], which could increase P and Fe availability to plants grown in both soil and hydroponic systems [25]. Furthermore, a SPAD meter measures leaf chlorophyll contents, and higher SPAD values indicate higher chlorophyll contents and potentially greater photosynthesis in the leaves. Our results (Figure 3) agreed with the results obtained in red lentil cultivar cv. Tigris, where PGPB increased total chlorophyll contents under both normal and Pb stress conditions [26]. Further studies about the mechanisms of lettuce growth promotion by IALR632 in hydroponic systems need to be performed with knockout mutations to determine which traits are specifically responsible for growth promotion [25]. Also important is how the inoculation of the bacterium affects native lettuce root microbial community and increases root health in hydroponic systems. There are only a few reports on the root microbiome of hydroponically grown crops [27], but numerous studies on root microbial community changes of land plants after bacterial inoculation [28,29]. For example, the rhizosphere of soilless-grown tomatoes has reduced bacterial and fungal diversity compared with soil-grown tomatoes [30].

The use of PGPB in agricultural production, particularly in controlled environment agriculture, has been greatly increasing because PGPB are naturally occurring, which makes them environmentally friendly [31]. Among PGPB, *Pseudomonas* spp. particularly receive attention because they can colonize plant roots well, produce various compounds including plant hormones, osmolytes and polysaccharides, and positively interact with other bacteria [31]. Our results also showed that IALR632 is able to colonize lettuce roots, translocate to leaves (Figure 7), and survive and proliferate inside host plants, which results in growth promotion [31]. The use of PGPB in hydroponically grown crops has the potential to further increase yields and food security, because the use of pesticides is highly limited in hydroponic systems. PGPB can keep plants healthier and help mitigate biotic and abiotic stresses. However, there are challenges to wide-scale use of biological products with a broad spectrum of plant growth promotion and disease prevention capabilities, such as factors influencing efficacy and biological commercialization bottlenecks. Currently, researchers have little knowledge about how to artificially construct functional and effective bacterial consortia, which are stable and are optimized to promote plant growth [25]. With respect to practical use and convenience, as well as formulation, individual PGPB that possess several functional plant growth-promoting traits are the best candidates currently. Single strains containing ACC deaminase activity, phosphate solubilization ability, and auxin production would be more successful in increasing yields in a wide range of crops [25]. In the future, more practical and efficient application methods of IALR632, such as drench, will be developed in hydroponically grown lettuce and spinach plants. Furthermore, the combination of this growth stimulant IALR632 and other biocontrol agents will be studied in controlled environment agriculture.

In summary, *Pseudomonas psychrotolerans* IALR632 possesses multiple functional plant growth-promoting traits and has been proven to promote plant growth in a broad spectrum of lettuce cultivars grown in hydroponic systems. It has the potential application to further increase lettuce yield in controlled environment agriculture.

## Figures and Tables

**Table 1:** The growth conditions in different hydroponic systems.

Systems	Dates	Tank Vol. (L)	No. of Plants	Temp. °C	Day/Night °C	LH	DLI	RH (%)
**GH-NFT**	12 April– 4 May 2021	90	36	20.2	21.9/18.6	8.7	25.5	63.3
**GH-NFT**	23 September– 21 October 2021	90	36	19.7	21.2/18.4	10.9	13.7	81.2
**IV-NFT**	31 March– 22 April 2022	260	144	20.2	20.9/19.3	18	16.2	58.8
**DWC**	26 January– 16 February 2022	18	18	17.5	18.6/13.3	5.4	13.2	36.3

GH-NFT: greenhouse NFT; IV-NFT: indoor vertical NFT; DWC: deep water cultivation; LH: light hours; DLI: daily light interval; and RH: relative humility.

## Data Availability

Not applicable.
